# Sustaining Brain Youth by Neural Stem Cells: Physiological and Therapeutic Perspectives

**DOI:** 10.1007/s12035-025-04774-z

**Published:** 2025-02-22

**Authors:** Matilde Santos, João A. Ferreira Moreira, Sónia Sá Santos, Susana Solá

**Affiliations:** Research Institute for Medicines (iMed.ULisboa), Faculty of Pharmacy, Universidade de Lisboa, 1649-003 Lisbon, Portugal

**Keywords:** Adult Neurogenesis, Aging, Brain Repair, Neural Stem Cells, Neurologic Disorders

## Abstract

In the last two decades, stem cells (SCs) have attracted considerable interest for their research value and therapeutic potential in many fields, namely in neuroscience. On the other hand, the discovery of adult neurogenesis, the process by which new neurons are generated in the adult brain, challenged the traditional view that the brain is a static structure after development. The recent findings showing that adult neurogenesis has a significant role in brain plasticity, learning and memory, and emotional behavior, together with the fact that it is strongly dependent on several external and internal factors, have sparked more interest in this area. The mechanisms of adult neural stem cell (NSC) regulation, the physiological role of NSC-mediated neuroplasticity throughout life, and the most recent NSC-based therapeutic applications will be concisely reviewed. Noteworthy, due to their multipotency, self-renewal potential, and ability to secrete growth and immunomodulatory factors, NSCs have been mainly suggested for (1) transplantation, (2) neurotoxicology tests, and (3) drug screening approaches. The clinical trials of NSC-based therapy for different neurologic conditions are, nonetheless, mostly in the early phases and have not yet demonstrated conclusive efficacy or safety. Here, we provide an outlook of the major challenges and limitations, as well as some promising directions that could help to move toward stem cell widespread use in the treatment and prevention of several neurological disorders.

**Graphical Abstract:**

Factors influencing neural stem cell plasticity and neurogenesis. Several intrinsic and extrinsic factors can modulate neural stem cells’ ability to change their fate and function and to generate new neurons in the brain. *CSF*, cerebrospinal fluid; *GCL*, granule cell layer; *SGZ*, subgranular zone. Created with BioRender.com

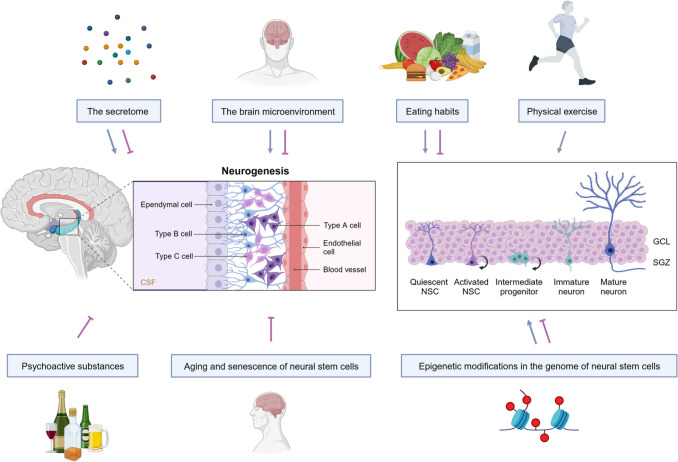

## Introduction

The term stem cells (SCs) was first introduced in the scientific literature in 1868 by the German biologist Ernst Haeckel, and since then, many definitions have come to light. SCs can be defined as units of organization of biological systems responsible for the regeneration and development of organs and tissues [1]. These cells have the unique potential of self-renewal as well as the differentiation to give rise to multiple cell lineages [2].

In the neurobiology field, SCs have been particularly relevant as they have helped researchers understand the nervous system’s development and function and foster novel therapies for neurological disorders. Here, we will first introduce some basic concepts of SCs, with a particular focus on neural stem cells (NSCs) — undifferentiated neural cells that can self-renew and differentiate into multiple neuronal and glial cell types of the central nervous system (CNS) [3, 4]. We will discuss the sources, characteristics, and applications of NSCs in neurobiology, including their crucial roles in brain development, plasticity, and repair, highlighting the process of neurogenesis. In addition, we will address how NSCs are influenced by environment, aging, and disease, as well as the potential use of induced pluripotent stem cells (iPSCs) derived from patient cells for the development of neurological disease models and therapeutic interventions. Finally, some of the recent challenges in the NSC field will be stated such as the pivotal need to ensure the safety and efficacy of SC-based therapies.

## NSCs and Adult Neurogenesis

### NSCs

NSCs are cells with two defining characteristics: the capacity to self-renew through cell division and the capacity to differentiate into all types of neural cells (multipotency), including neurons, astrocytes, and oligodendrocytes [5–7]. In mammals, NSCs are found in discrete regions of the adult brain. These spatially restricted brain areas comprise multicellular microenvironments and are termed neurogenic niches, supplying NSCs with the necessary factors to maintain stem cell self-renewal and fate-committed asymmetrical division. Although non-canonical neurogenic niches have already been identified [8], the two main neurogenic regions in mammal brains are the subventricular zone (SVZ) abutting the lateral ventricles, where new neurons are generated and then migrate through the rostral migratory stream (RMS) to the olfactory bulb (OB) to become interneurons; and the subgranular zone (SGZ) in the dentate gyrus (DG) of the hippocampus, where newborn neurons migrate into the granule cell layer and become dentate granule cells [9, 10]. In the basal cell side, SVZ-derived NSCs are known to interact with the basal lamina from the local vasculature through basal processes [11–13]. In fact, through cell projections, they are capable of sensing different factors, such as cytokines, neurotransmitters, hormones, or growth factors, that regulate their fate and activity [11, 14]. The generation of NSC-derived new neurons in these described regions is followed by the integration of these newborn cells into pre-established circuits. Briefly, newborn neurons undergo a lengthy process of morphogenesis, including the *de* *novo* growth of axons and dendrites and the formation of synapses. In the adult SVZ region, neurogenesis is thought to contribute to fine olfactory discrimination, odor-reward association, and olfactory learning and memory [15, 16]. However, several studies have reported differences in SVZ neurogenesis between rodents and humans. In the human brain, for instance, immature neurons generated in the lateral ventricle wall do not migrate to the olfactory bulb but instead to the striatum region [17]. In this region of the human brain, the biological function of human SVZ-derived NSCs is still largely unclear. On the other hand, although most studies reporting a role for adult neurogenesis and stress resilience have been performed in hippocampal neurogenic niches, it has already been demonstrated that the adult SVZ neurogenic process is also compromised under unpredicted prolonged stress [18, 19]. Curiously, it has been recently shown that both anxiety- and depression-like states lead to marked olfactory deficits along with impaired adult neurogenesis [20].

Regarding hippocampal adult NSCs, the new cells were already shown to have enhanced excitability and plasticity before they were fully integrated into the brain circuitry. After their integration, they are thought to contribute to learning, memory, and pattern separation [6, 21], as well as to stress response and emotional regulation [22]. Interestingly, it has been shown that the experiences of animals, especially those occurring during the maturation of hippocampal newborn neurons, can deeply influence the responsiveness of these cells when they become fully mature. The main hypothesis emerging from several computational studies is that adult newborn neurons allow plasticity by preserving the information that is contained in mature hippocampal neurons. However, a more recent model further proposes that these immature neurons might serve as a pattern integrator by linking events that occur closely in time [23]. Thus, it appears that this process of cell-level renovation is not fixed or simply restorative, but it represents an adaptive response to deal with different challenges throughout life [24]. All in all, despite the insertion of molecular, synaptic, or morphological changes at individual cells, the addition of new neurons to the hippocampal area appears to facilitate the process of making functional alterations in its neural circuitries. Understanding the precise role of adult NSCs, immature NSC-generated cells, and their contribution to neuroplasticity is, therefore, a vibrant area of research with broad implications for human brain health that are far to be completely clear.

### Adult Neurogenesis

Neurogenesis, the process of generating functionally integrated neurons from progenitor cells, was traditionally believed to only occur during embryonic and early postnatal development in the mammalian CNS. However, in the 1960s, under the central assumption that the adult mammalian brain would not generate new neurons, J. Altman and G.D. Das reported, for the first time, the existence of newborn neurons in different structures of adult rats, such as the hippocampus and neocortex [25, 26]. At that time, the existence of dividing cells in the adult mammalian brain was only detected with a routine technique to study development in rodents, in which [3H]-thymidine is incorporated into the DNA of dividing cells [27]. Subsequently, new, improved techniques for visualizing neural cells helped to crumble the dogma of the absence of newborns in adult mammalian brain neurons [28], either by staining markers of proliferation [29] and neurogenesis in post-mortem human brain tissue [30] or other techniques, such as by radiocarbon dating methodologies [31].

Although some skepticism around this type of brain regeneration has always persisted, neurogenesis has been considered across the last decades as a normal physiological mechanism of human neural plasticity and cognition throughout life. Nevertheless, the idea of adult neurogenesis in humans has always been a controversial issue in the field of neuroscience. In 2018, a polemic study questioned the extent of adult-generated neurons across the human lifespan [32]. This study was later revealed to be based on several experiments with technical weaknesses and misinterpretations. Other convincing and elegant studies have then reported the existence of human adult neurogenesis and its important role in sustaining cognitive function throughout life, also revealing a strong link between its decline and compromised cognitive-emotional resilience [33–36]. Notably, another polemic study reappeared, questioning the existence of adult hippocampal neurogenesis in humans [37]. In this study, the authors observed transcriptomic signatures of adult neurogenesis in mice, pigs, and monkeys but not in human hippocampal tissue. Indeed, the poor characterization of both human neural precursor markers and their precise molecular transitions in different layers of the human cortical structure have enormously contributed to the difficulty of detecting adult neurogenesis in humans. Most of these studies have largely relied on detecting doublecortin by immunostaining. This protein is an immature neuron marker that requires complex and tricky histological protocols for post-mortem adult human brain detection. Still, the idea of the existence of lifelong neurogenesis in the human hippocampus is not excluded. Several accurate and robust studies have later demonstrated the existence of adult human hippocampal neurogenesis, including also a more recent study describing the molecular landscapes of human hippocampal immature neurons across the lifespan [38]. In this study, the authors used a more sophisticated method, i.e., a single-nucleus RNA sequencing assisted by a machine learning-based analytical approach, to evaluate human immature neurons more precisely across the lifespan. They also explained why conventional unsupervised methods, such as those recently published by D. Franjic and collaborators [37], were insufficient to identify immature neuronal populations.

**Fig. 1:**
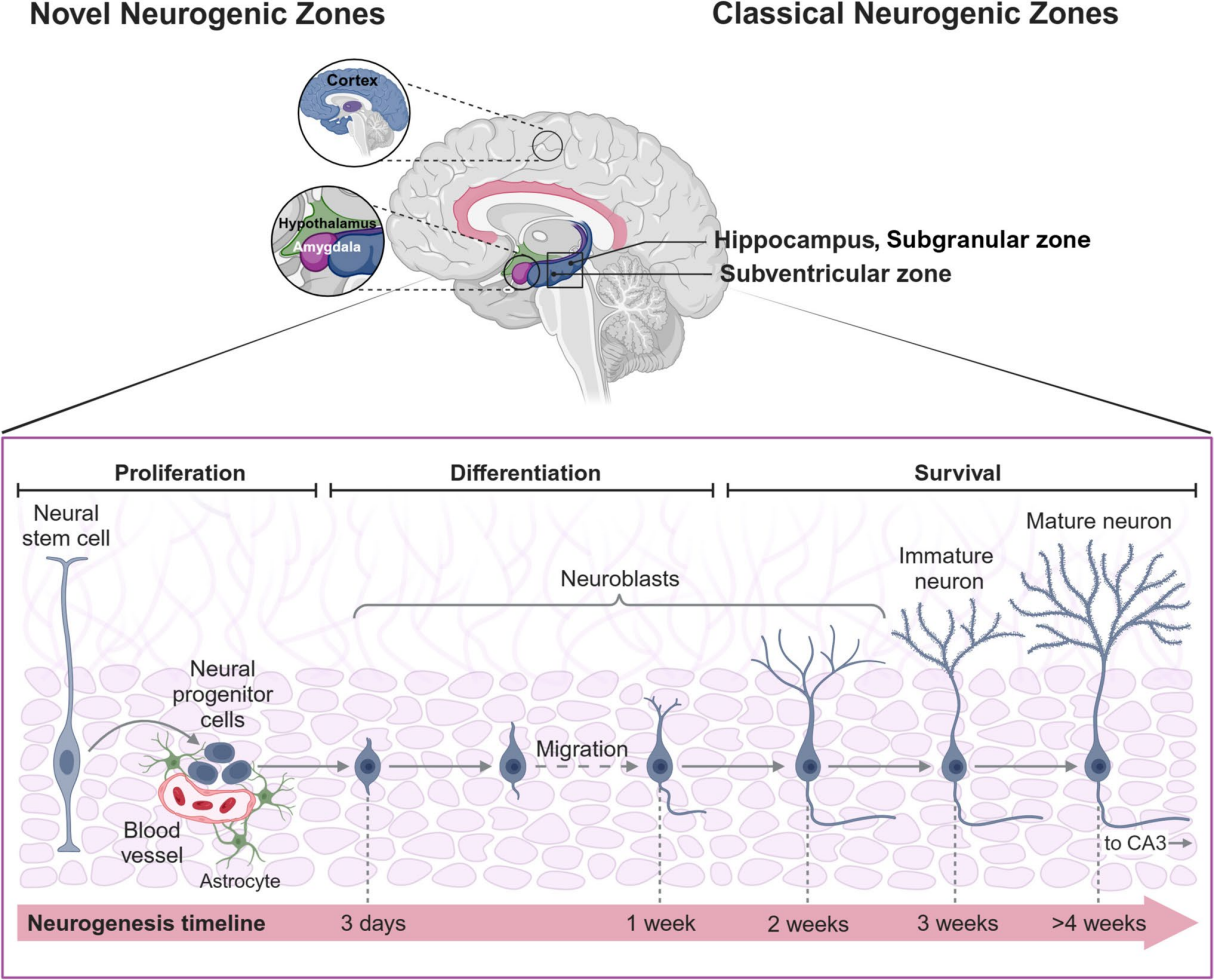
The “novel” and “classical” adult neurogenic zones, as well as the major cellular stages of neurogenesis, depicted schematically. Schematic representation of the 10 different brain regions, classic – the hippocampus and subventricular zone – and “novel” – the prefrontal cortex, hypothalamus, striatum, amygdala, and piriform cortex – where progenitor cells are generated. Experiments have shown that progenitor cells can stray from the conventional path in the DG and SVZ and differentiate and mature in diverse brain regions, including the prefrontal cortex, striatum, substantia nigra, and amygdala, which are referred to as “novel neurogenic zones.” *CA3*, cornu ammonis 3 (region of the hippocampus). Adapted from [213]. Created with BioRender.com

The neurogenic process includes several phases, including cell proliferation, neuronal fate specification, migration, differentiation, and survival of newly generated neurons, as well as functional (i.e., synaptic) integration into existing neural circuits (Fig. 1) [39, 40].

As already mentioned, in the SGZ, adult neurogenesis is thought to play a role in cognition, including memory and spatial navigation, but also in mood regulation (i.e., affective behaviors). Likewise, particularly in rodents, adult neurogenesis in the SVZ/OB is thought to contribute to optimal olfactory circuit formation [39]. In the “classical” neurogenic zones, heterogeneous progenitor cells will give rise to the new neurons. During the precursor cell phase, NSCs from the SGZ, also called type-1 progenitor or radial-glia-like cells, divide asymmetrically to give rise to type-2 cells, transit amplifying neural progenitors [39, 41]. After this stage, the fate of the neural progenitor is determined. Type-2 cells are highly proliferative and already committed to a neuronal lineage, dividing symmetrically to generate type-3 cells (neuroblasts). These intermediate progenitors will then originate glutamatergic granule neurons, which in turn will migrate into the granule cell layer of the DG, where they will fully maturate [41]. Their maturation generally mirrors a slower time scale of the neuronal maturation process observed in development [4]. The SVZ region also contains different cell types. First, the slow-diving type B cells generate fast-diving type C cells. These cells give rise to type A cells (neuroblasts), which then migrate tangentially via the RMS to the OBs, where they become fully mature neurons [41].

While the biological function of adult neurogenesis in humans is still under debate, *in* *vitro* approaches are also emerging to develop neural cell types from human neuroblasts as potential therapeutic strategies for introducing regeneration exogenously in degenerating brain regions [42, 43]. Noteworthy, the presence of NSCs in the adult human brain is unquestionable, and new evidence has suggested that neural progenitor/stem cells also regulate their surroundings through paracrine signaling [44]. In fact, their location in the brain, close to the vasculature and the cerebrospinal fluid-filled ventricles, places these nutrient-sensing cells in an ideal position to receive extrinsic cues from the environment and relay them to the members of the neurogenic niches [11]. Therefore, beyond their involvement in full differentiation and neural tissue replacement, human endogenous NSCs could also act as master regulators of neuroregeneration and plasticity through mechanisms not completely resolved.

### The Pivotal Role of the Neurogenic Niche

The SVZ and SGZ neurogenic niches facilitate and support neurogenesis through different secreted factors and cell–cell contacts. These, in turn, maintain NSCs in a quiescent and undifferentiated state capable of preventing their depletion or directing their differentiation in appropriate lineages [45].

But what are the exact mechanisms by which these niches support and regulate neurogenesis? In the adult SVZ neurogenic niche, the choroid plexus appears to have a crucial regulatory role. In fact, this highly vascularized tissue secretes the cerebrospinal fluid (CSF, a rich source of proteins, lipids, hormones, cholesterol, glucose, microRNAs, and many other molecules and metabolites crucial for brain functions, but also for NSC maintenance and proliferation) [46, 47]. In SVZ, NSCs are in direct contact with the CSF-filled ventricles and, thus, receive the appropriate cues that allow them to sense external changes. These include key signaling molecules that influence neurogenesis, such as the fibroblast growth factor (FGF-2), bone morphogenic proteins, and insulin-like growth factor 2 [3, 46]. Curiously, an interesting discovery also revealed that the choroid plexus regulates the number of NSCs through the release of microRNA-204 [48]. This microRNA then reaches the NSCs in the SVZ, where it regulates the expression of genes involved in the cell cycle, migration, and differentiation of NSCs. Furthermore, microRNA-204 prevents NSCs from becoming prematurely activated and differentiated [48]

Strikingly, the CSF can also control the NSC behavior by hydrostatic forces. As an example, the multiciliate ependymal cells that form the ventricular epithelium [49] generate a unidirectional CSF flow that induces gradient guidance cues capable of promoting the migration of neuroblasts along the RMS. Indeed, the ependymal cells were also shown to regulate the neurogenic niche, as well as the number and the lineage fate of NSCs, through the release of both Noggin [50] and the ependyma-derived matricellular protein cellular communication network factor 1 [51].

The microglia cells are other components of the neurogenic niche pivotal for the process of neurogenesis in the SVZ region. Microglia are the primary immune cells of the brain, having a wide range of functions, varying from phagocytosis to neuroprotection [4]. Interestingly, in the SVZ areas, these cells have a different morphology, which may provide them with specific properties and roles in the regulation of SVZ neurogenesis [52]. They modulate neurogenesis by releasing soluble factors, extracellular vesicles, and gap junctions. In contrast with what occurs in the SGZ niche of the hippocampus, SVZ microglia do not phagocyte neuroblasts, providing them with trophic support to induce their survival [52]. In fact, in the adult DG neurogenic niche, these types of cells are found uniformly distributed in the hilus and around the border of the GC layer [53]. Microglia contributes, therefore, to the homeostasis of neurogenesis not only via phagocytosis of differentiated newborn neurons that have undergone apoptosis but also by providing trophic support via cytokine release. Changes in the environment activate microglial cells, altering their morphology and the pattern of secreted factors [54]. Thus, it is the equilibrium between pro-inflammatory and anti-inflammatory signaling that will dictate whether the environment supports or prevents adult neurogenesis by microglia [54]. As an example, tumor necrosis factor-α and interleukin-6, two of the main pro-inflammatory cytokines secreted by traditionally activated microglia, can favor astrocytic-lineage specification of NSCs [55]. In fact, individual cytokines display pro-neurogenic or anti-neurogenic properties based on the environment [4]. As an illustration of this dual regulatory effect, the cytokine transforming growth factor-β (TGF-β), which is secreted by microglia and activates both pro-inflammatory and anti-inflammatory pathways, has been shown to either induce [54] or inhibit [56] neurogenesis [4]. Indeed, Battista and colleagues [54] found that more activated microglia resulted in additional neurogenesis and TGF-β levels in the DG. They also showed that blocking TGF-β with an antibody reduced new neurons in the DG and that TGF-β induces neurogenesis in both human-induced pluripotent stem cells (hiPSCs)-derived NSCs and 3D neural models [54]. On the other hand, Buckwalter and colleagues [56] reported that astrocyte-delivered TGF-β1 caused chronic inflammation capable of impairing neurogenesis and blood vessel formation in the aged brain. The authors used transgenic mice overexpressing astrocytes-delivered TGF-β1 and found fewer new neurons and more vascular problems in the hippocampus in these mice, rescued by blocking TGF-β [56].

Importantly, astrocytes are highly specialized glial cells responsible for trophic and metabolic support of neurons [57]. In the SVZ neurogenic niche, they contribute to the establishment and maintenance of the neurogenic microenvironment by promoting the proliferation and neuronal fate commitment of NSCs [57]. In addition, astrocytes unsheathe chains of neuroblasts, also playing a fundamental role in the neuroblast migration along the RMS to the olfactory bulb [58, 59]. Undoubtedly, astrocytes represent the major contributors to neurogenic niche regulation, having an active role in the regulation of neurogenesis derived either in SVZ or DG neurogenic niches [46]. These cells not only promote the proliferation and differentiation of NSCs into mature GCs [60] but also support the integration of the newborn neurons through the vesicular release of D-serine [61], an endogenous agonist that activates N-methyl D-aspartate receptors in the brain [62]. Furthermore, astrocytes direct NSCs toward a neurogenic fate via a wide range of secretory factors and signaling molecules, such as neurogenesin-1 and Wnt3 [63].

Another crucial and integral constituent of the SC niche is its vasculature network. Importantly, neurogenic regions are more vascularized than other non-neurogenic regions of the brain, and the blood vessels are more permeable due to a lack of blood vessel coverage by astrocyte end feet and pericytes [59, 64]. The blood flow is also slower in these regions [65]. All these characteristics suggest that neurogenic niches, including NSCs, are the brain regions with the easiest access to blood-derived signals to the cells. Moreover, endothelial cells secrete soluble factors that promote changes in NSC fate and direct cell–cell interactions [45].

Finally, adult neurogenesis may also be modulated in neurogenic regions by a wide range of local neurotransmitters [66]. Hippocampal neurogenesis in the adult SGZ is particularly sensitive to the surrounding neuronal activity in the area [46]. For example, the gamma-aminobutyric acid (GABA) neurotransmitter released from hippocampal neuroblasts provides a feedback mechanism to control the proliferation of NSCs, namely by activating GAB ${A}_{A}$ receptors [67]. Although through distinct pathways, both type-1 and type-2 cells in the SGZ respond to GABA, the main inhibitory neurotransmitter in the brain. Curiously, type-1 cells became quiescent by GABA, while type-2 cells became activated and responsive to active GABAergic inputs, undergoing neuronal differentiation [68] and synaptic integration of the young newborn neurons [69]. Hence, local GABA appears to suppress proliferation while inducing differentiation and survival. Another neurotransmitter required for optimal survival of NSCs is glutamate, the principal excitatory neurotransmitter in the brain. Glutamate can modulate neurogenesis by affecting the proliferation, differentiation, migration, and integration of new neurons in both the SVZ and the SGZ [4]. However, different aspects of the neurogenic process appear to be regulated by modulatory neurotransmitters.

**Fig. 2:**
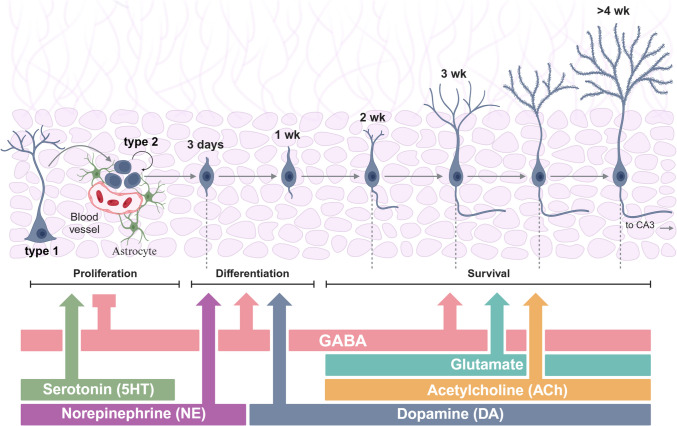
Regulation of the neurogenesis by local circuit factors. All stages of neurogenesis (proliferation, differentiation, and survival) are targets of regulation by network factors. Local GABA appears to suppress proliferation while inducing differentiation and survival. Glutamate is necessary for proper survival as well. Modulatory neurotransmitters appear to regulate different parts of the process; for instance, serotonin induces proliferation, whereas acetylcholine is necessary for proper maturation and survival. The effects of the other major neuromodulatory systems [norepinephrine, dopamine] on neurogenesis are less well understood. *CA3*, cornu ammonis 3 (region of the hippocampus); *GABA*, gamma-aminobutyric acid; *5HT*, serotonin; *ACh*, acetylcholine; *NE*, norepinephrine; *DA*, dopamine. Adapted from [4]. Created with BioRender.com

For example, serotonin promotes NSC proliferation in both the SVZ and the SGZ, but it also enhances neuronal differentiation and survival in the SGZ [4]. Acetylcholine, in turn, is required for appropriate maturation and survival of new neurons in the SGZ, as well as for their integration into the existing hippocampal circuitry, while dopamine has a complex and region-specific role in neurogenesis. Interestingly, dopamine was shown to stimulate NSC proliferation and neuronal differentiation in the SVZ, but it inhibits these processes in the SGZ. Noradrenaline, on the other hand, enhances NSC proliferation and neuronal differentiation in both the SVZ and SGZ, but it also regulates the migration of neural progenitor cells (NPCs) from the SVZ to the olfactory bulb, where they mature into interneurons (Fig. 2) [70].

As well-known, modulatory neurotransmitters are involved not only in the regulation of neurogenesis but also in the regulation of several cognitive, emotional, and behavioral functions that depend on the activity of the newly generated neurons. For example, serotonin, acetylcholine, and noradrenaline are implicated in learning and memory processes that require hippocampal neurogenesis. Dopamine, serotonin, and noradrenaline are all also associated with mood disorders, such as depression and anxiety, which in turn are also linked to impaired neurogenesis. Glutamate and GABA are key in regulating synaptic plasticity and network oscillations, which again are influenced by the integration of new neurons. Therefore, modulatory neurotransmitters have a dual role in the adult brain. While controlling the formation and function of new neurons, they also mediate the effects of these neurons on brain function and behavior [70].

Neurotransmitters, growth factors, and membrane-associated ligands are just a few of the signaling molecules that have been found to control NSC activity in the neurogenic niche [3]. Most of these molecules operate in a paracrine, non-cell autonomous manner and are derived from different cellular elements of the NSC niche [3]. However, it is still unclear whether NSC fate can also be influenced by the molecules they produce. In 2018, Zhou and colleagues demonstrated that NSCs in the SGZ can control their quiescence in an autocrine manner. This study showed the significance of NSCs for the development of their own niche [71].

Since several neurodegenerative, neurodevelopmental, and injury-based diseases are associated with adult neurogenesis impairments [72], a further understanding of the regulatory processes on the microenvironment of the neurogenic niches appears to be crucial for improving NSC-driven brain regeneration and accelerating their use and success in the treatment and prevention of neurological disorders [3].

## NSC Dysfunction in Aging

Most tissues gradually lose their ability to regenerate throughout life, leading to functional decline and inadequate healing after damage and illness [73]. Like other tissues of the body, the brain also deteriorates with age [74]. Being primarily composed of postmitotic cells, the brain tissue is even more sensitive to several age-dependent changes [75]. Therefore, preserving a healthy NSC population in the brain throughout life is crucial for (1) maintaining the homeostatic potential of neural regeneration and brain health and (2) preventing the incidence of brain tumors in the elderly [74].

As already mentioned, the levels of neurogenesis are positively and negatively affected throughout the life of mammals by external factors, such as neuroinflammation, physical activity, diet, environment stimuli, learning, abuse of psychoactive substances, stress, depression, antidepression medication, and aging, among others [4, 76]. One of the most important negative influences is aging, which is deeply associated with a sharp drop in the NSC pool, as well as with self-renewal and differentiation potentials [77]. Indeed, a weaker performance on learning and memory tasks and reduced olfactory discrimination are some consequences of this drop [74]. Several human studies have already revealed a significant age-associated decline in the number of intermediate progenitors and proliferating cells in the DG region [78, 79], remaining mostly microglia cells in this region [80]. In addition to the NSC number decline throughout aging, there is an increase in the time they spend in quiescence. Furthermore, aged NSCs favor a self-renewal-associated symmetrical division to the detriment of a neurogenesis-associated asymmetrical division [79, 81]. Regardless of whether the decline involves the number of the functionality of NSCs, which is influenced by both external and internal changes in NSC signaling, the ultimate consequence is a reduction in neurogenesis [74, 82, 83].

Therefore, understanding the processes underlying SC aging holds great promise for developing efficient methods to sustain and increase the potential of SCs for tissue regeneration as we age. Numerous cellular processes may be involved in the decline in the number and/or function of NSCs during aging, including the intrinsic suppression of NSCs proliferation and maturation, the lack of trophic support and deficits in the neurogenic niche, the decline in neuronal fate commitment, the decrease in NSC self-renewal, and ultimately NSC death [73, 74]. Some of these age-associated alterations could be targeted to delay the effect of aging in NSCs.

### Metabolism in Aging

One of the intrinsic changes that comes with age is an altered cell metabolism. In this regard, it has been shown that SC metabolism is critical for their homeostasis, maintenance, and cellular fate. In addition, it has been shown that age-induced metabolic decline strongly affects NSCs’ behavior, including their ability to differentiate and proliferate. The fundamental homeostatic processes affected include nutrient sensing, mitochondrial maintenance, and proteostasis — each recognized as a hallmark of aging [84]. Of note, the survival of every organism depends on its capacity to sense and make use of environmental nutrients for its development and growth. The nutrient-sensing pathways will either activate homeostatic processes, such as the mobilization of internal supplies, or engage in anabolism and storage, depending on whether there is a shortage or an abundance of nutrients, respectively [85].

Interestingly, under nutrient-deprived conditions such as caloric restriction, metabolism shifts toward a state dominated by ketosis, which is suggested to contribute to enhanced longevity [86]. The best-characterized pathways implicated in NSC regulation, nutrient sensing, and aging are the insulin and insulin/insulin-like growth factor-1 signaling, the mechanistic target of mammalian target of rapamycin (mTOR) signaling pathway, and the Sirtuins signaling pathway [87, 88]. In fact, in both human and animal models, numerous studies have claimed that dietary intervention can have lifespan-extending effects by the modulation of these pathways [89]. For example, Fontana and collaborators demonstrated how caloric restriction can lengthen lifespan in mice, namely, how it may also have neuroprotective benefits in humans [90]. Indeed, caloric restriction has been shown to have beneficial effects on NSCs and neurogenesis by activating the Sirtuin pathway, which enhances NSC survival, proliferation, and differentiation [91]. It can also inhibit the mTOR pathway, which suppresses NSC quiescence, senescence, and inflammation. Moreover, caloric restriction can modulate the insulin/IGF-1 pathway, leading to higher expression of antioxidant systems and, therefore, impacting NSC metabolism, neurogenesis, and cognition [86, 91].

However, not all dietary interventions have positive effects on NSCs and neurogenesis. Overnutrition, which is a condition of excessive intake of calories and nutrients, can have detrimental effects on NSCs and neurogenesis. It impairs the Sirtuin pathway and activates the mTOR pathway, having the opposite effects of caloric restriction. Furthermore, overnutrition can dysregulate the insulin/IGF-1 pathway [91]. Thus, nutrient sensing pathways play an important role in NSC aging, as they mediate the effects of diet and metabolism on NSC function and fate. Molecular regulation of these pathways appears to represent a good strategy to delay or reverse some of the age-related changes in NSCs and their niches and improve brain health and function.

As mentioned previously, mitochondrial dysfunction is considered to be one of the hallmarks of aging, contributing significantly to age-associated neurogenesis deficits [84, 92]. Mitochondria are multifunctional organelles that have several important functions in the cell, ranging from cell signaling to bioenergetics [92]. To understand their significance in NSC fate, it is important to note that during NSC differentiation, their metabolism shifts from being mainly glycolytic to a progressive dependence on mitochondrial oxidative phosphorylation (OXPHOS) [93]. Accordingly, mitochondria also experience morphological changes as NSCs commit to a neuronal lineage, balancing between fission and fusion during the NPC phase and then elongating upon differentiation into neurons [92]. Furthermore, mitochondria are morphologically different in active or quiescent NSCs. Depending on their current state, quiescent or active, and proliferating, NSCs have different requirements for protein and metabolic homeostasis [92]. Mitochondria are, therefore, thinner and elongated in intrinsically glycolytic quiescent NSCs but globular or tubular in activated NSCs, where the metabolic swift to mitochondrial OXPHOS occurs to support the new energy demands of the cell [92]. Curiously, the age-associated changes in the metabolic balance between glycolysis and OXPHOS have been shown to favor a more dormant state [84]. Indeed, an age-related increase of glycolytic enzymes is observed, whereas downregulation of nuclear-encoded electron transport chain proteins also occurs. In addition, consistent with a shift towards glycolytic metabolism, aged NSCs show considerable losses in mitochondrial content, membrane potential, ATP generation, and oxygen consumption, all of them correlated with decreased NSC proliferation [94]. Therefore, as both activated and quiescent NSCs rely on mitochondria to manage metabolism, improving mitochondrial function in the elderly may also be an efficient manner to improve cellular performance across the NSC lineage, resulting in an increase in neurogenesis overall [74]. One of the key regulators of mitochondrial function and biogenesis is the peroxisome proliferator-activated receptor γ coactivator α (PGC-1α), which is also involved in the modulation of NSC fate and aging [95]. Several recent studies have suggested that PGC-1α can protect NSCs from oxidative stress, promote their differentiation into neurons and glia, and prevent or delay age-related neurodegeneration [96]. For example, a study by St-Pierre et al. [97] revealed that PGC-1α overexpression in NSCs increased their resistance to oxidative stress and enhanced their neuronal differentiation in vitro and in vivo. Moreover, Cui et al. [98] reported that PGC-1α deficiency in NSCs impaired their neurogenic potential and increased their susceptibility to amyloid-β toxicity, a hallmark of Alzheimer’s disease. Conversely, activating PGC-1α by resveratrol improved NSC function and ameliorated cognitive deficits in a mouse model of Alzheimer’s disease [98]. Finally, He et al*.* show that increased NSC expression of PGC-1α in a monkey model of Parkinson’s disease (PD) may play a role in NSC-mediated reduction of neuronal damage [99].

### Proteostasis in Aging

Protein homeostasis, the delicate balance of cellular protein levels, is also a pivotal aspect of the NSC fate decision [100]. Precise regulation of protein synthesis, folding, conformational stability, and degradation is necessary to maintain cellular proteostasis and to prevent protein aggregation and misfolding [101, 102]. Importantly, several pathological conditions linked to old age, such as neurodegenerative diseases, have been associated with the loss of proteostasis and the consequent accumulation of protein aggregates, suggesting that the capacity of the proteostasis network diminishes throughout age [102]. The appearance of misfolded and neurotoxic proteins is also accelerated by this decrease [103].

On the other hand, quiescent versus active NSCs depend on different proteostatic mechanisms. The first relies mainly on the lysosome-autophagy pathway, while active NSCs depend on molecular chaperones and the proteasome proteolytic system [74, 104]. Normally, quiescent NSCs contain protein aggregates in their enlarged lysosomes; however, that accumulation increases with age [74, 105]. One interesting observation was the existence of these aggregates in higher levels in old quiescent NSCs isolated from the SVZ in comparison with the young ones [74]. In fact, this evidence confirms a decline in lysosome-autophagy function in these cells. The deterioration of this function, on the other hand, results in a reduction in their ability to become active and proliferate [104]. Of note, it has been shown that the induction of the lysosome-autophagy system by overexpression of a key transcription factor, as the transcription factor EB, is sufficient to enhance NSC activation in the aged brain and exit their quiescent state [74]. This clearly indicates that the dysfunction of this system is a major factor for aging-associated quiescent NSC decline and could be a good pharmacological target to avoid the aging effects of NSC [106].

### Dysbiosis in Aging

It is well known that gut microbiota, the community of microorganisms that live in the intestines, has a functional relationship with neurogenesis through the gut–brain axis. These microorganisms produce several neurotrophins and neurotransmitters that are vital for the survival and differentiation of NSCs [107]. Moreover, it has been established that alterations in the gut microbiome’s composition and metabolite production, namely, influenced by dietary changes, can significantly impact neurogenesis. For example, a diet high in fat can lead to dysbiosis, resulting in an increased production of short-chain fatty acids like propionate and butyrate. These changes also lead to an increase in reactive oxidative species and mitochondrial activity in NSCs, inducing premature differentiation and depletion of the NSC pool in the adult neurogenesis niches of mice fed with a high-fat diet. [108]

Age-related dysbiosis is a significant contributor to the global inflammatory state in the elderly, also known as “inflammageing.” Indeed, in younger individuals, the gut microbiota tends to be more diverse compared to their older counterparts, who exhibit less diversity and a high portion of disease-causing microbes [109]. This shift towards a pro-inflammatory microbial community inhibits the growth of beneficial commensal bacteria, leading to chronic inflammation [110]. In fact, the microbiota scenario in aging compromises the integrity of the intestinal epithelium and escalates the permeability of the gut, allowing opportunistic bacteria and endotoxins to migrate into the bloodstream and triggering a series of inflammatory responses that enhance the risk of developing aging-associated pathologies [111]. Herein surges a promising opportunity to interfere, mitigate, or even reverse some of the microbiota changes in aging. Thus, the discovery of associations between the gut microbiome and diseases prevalent in the elderly paves the way for innovative interventions, as by manipulating the microbiome, we could potentially ameliorate or even prevent neurological diseases [112]. Adult neurogenesis could represent one of the potential areas of intervention.

### Epigenetic and Senescence Changes in Aging

To produce new neurons, NPCs need coordinated changes in their pattern of gene expression, mainly controlled at the level of gene transcription [113]. Epigenetic mechanisms play an important role in this control, being involved in NSC self-renewal, fate specification, maturation, and, consequently, adult neurogenesis regulation [113, 114]. The loss or accumulation of covalent histone modifications, DNA modifications (e.g., methylation), and global alterations to chromatin conformation occur naturally through aging [75, 105] and necessarily affect the expression of genes involved in the multipotency and differentiation of NPCs.

On the other hand, one of the consequences of epigenetic changes is cellular senescence, a homeostatic process that prevents the accumulation of damaged cells and neoplastic transformation [115]. In this stress-induced state, cells remain in a stable cell cycle arrest and their capacity to proliferate diminishes [75, 115]. Senescent cells display phenotypic changes including morphological, metabolic, and epigenetic alterations that immanently change their normal functions [105, 106]. Noteworthy, one of those modifications takes place in their secretome, where upregulation of pro-inflammatory molecules results in the secretion of pro-inflammatory proteins, chemokines, interleukins, proteases, and growth factors, known as the senescence-associated secretory phenotype [106].

Cells that feature a high level of damaged DNA, characteristic of aged cells, become senescent, with reduced proliferation [75]. As in other organs, mechanisms for DNA repair are reduced in aged brains, and DNA damage starts to accumulate, increasing energy demands and reactive oxygen species levels [75]. Cells with features of senesce have been identified in the aging brains of both rodents and humans, particularly in the context of neurodegenerative disease [116]. These cells disturb the milieu of the NSC niche, impacting the surrounding cells, which imminently results in age-associated dysfunction [116].

#### NSC in Brain Injury and Potential Therapies

As already stated, the neurogenic niches are dynamic environments that require tight regulation for the maintenance of their homeostasis and support of the adult neurogenesis process. Conditions that shift their state of equilibrium first trigger an adaptive response, in which NPCs become activated and exhibit higher levels of proliferation and differentiation outside of the regular lineage programming [72].

**Fig. 3:**
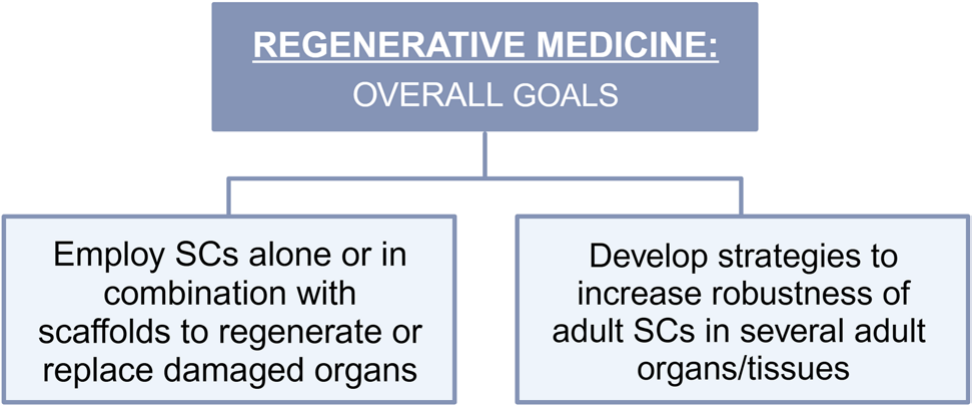
Overall goals of regenerative medicine. Regenerative medicine is a field of research that aims to restore or replace damaged human cells, tissues, or organs and to positively control the adult stem cell compartment so that stem cells remain robust throughout adulthood to improve health and quality of life. *SCs*, stem cells. Adapted from [123]. Created with BioRender.com

A neurological disease occurs when an event is so severe that adaptive responses can no longer sufficiently restore the disrupted balance. This results in the loss of homeostasis, leading to permanent physiological malfunction [117]. As an example, after the onset of an ischemic stroke, a hypoxic environment is created in the area of injury due to the hypoperfusion of the brain tissue. This environment stimulates the proliferation and migration of NSCs to the stroke area, which is called stroke-induced neurogenesis [118]. In this regard, abnormalities in adult neurogenesis have been linked to demyelinating, inflammatory, neurodegenerative, and injury-based disorders [76, 119]. Accordingly, therapeutic interventions involving NSCs have been shown to improve several pathological brain phenotypes, as summarised in (Fig. 3) [119].

**Fig. 4:**
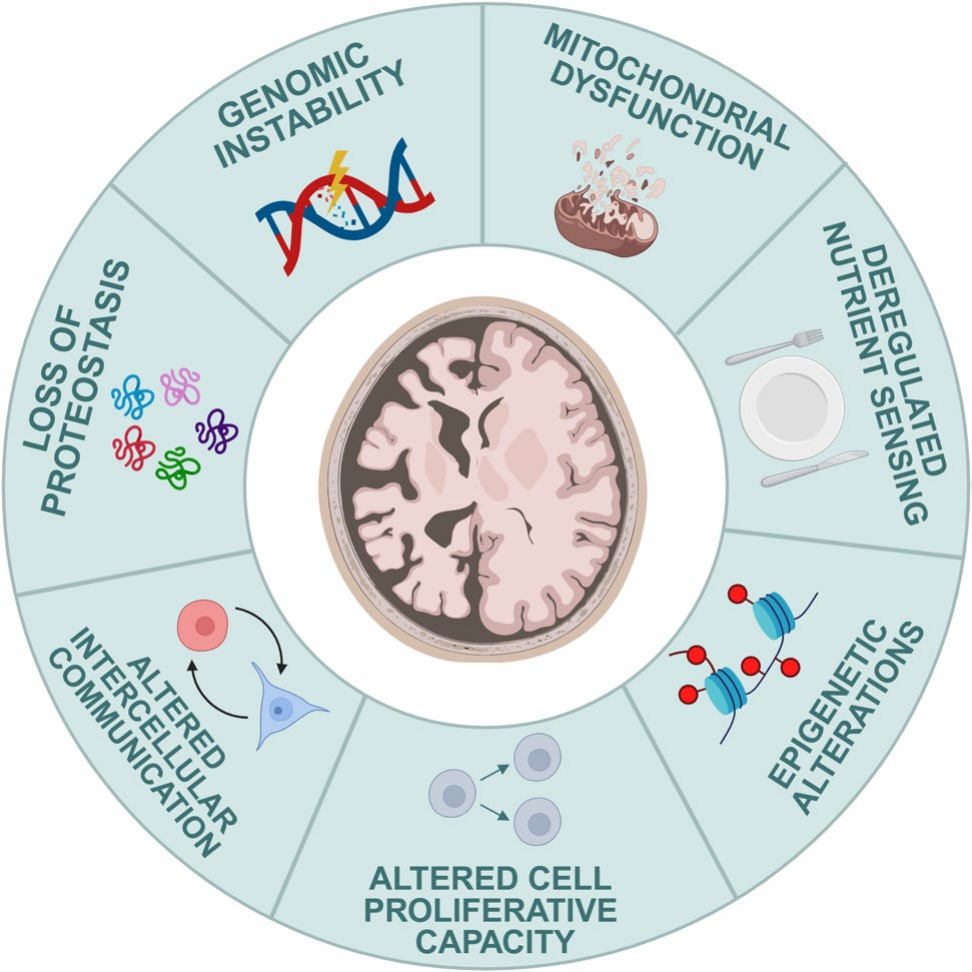
Molecular and cellular associated with neural aging. Several basic homeostatic functions in the brain become altered through aging. These include genomic instability, mitochondrial dysfunction, deregulated nutrient sensing, epigenetic alteration, altered cell proliferative capacity and intercellular communication, and loss of proteostasis, among others. Adapted from [214]. Created with BioRender.com

Regenerative medicine comprises the emerging field of medicine dedicated to the development of methods to replace, engineer, or regenerate human cells, tissues, and organs [120]. In this regard, advanced regenerative therapies have recently relied on the generation and use of therapeutic SCs for either a direct application in damaged tissues or for tissue engineering [1, 120–122]. The first successful transplantation of hematopoietic SCs dates more than half a century ago. That achievement encouraged clinicians to apply adult SCs in several medical problems, such as myocardial infarction, stroke, spinal cord injury, macular degeneration of the retina due to aging, diabetes, Alzheimer’s disease, liver injury, and Parkinson’s disease [76, 123–134]. However, the results for other clinical applications, besides hematological applications, have not been all that promising [135]. The primary objectives of regenerative medicine have been to utilize stem cells in critical emergency scenarios and to enhance adult stem cell niches, preserving their resilience throughout adulthood (Fig. 4) [76, 123].

SCs can be obtained from four main sources: (1) embryonic tissue; (2) fetal tissues, such as the fetus, placenta, and the umbilical cord; (3) certain tissues from the adult organism, e.g., fat, bone marrow, skeletal muscle, skin, or blood; and finally from (4) differentiated somatic cells after their genetic reprogramming in iPSCs [1]. Thus far, the most used SCs in cell therapies and tissue engineering procedures have been SCs derived from adult tissues. These cells are safer, do not bring the mutational and other adverse effects associated with pluripotent cells, and do not raise ethical and legal issues, such as the ones obtained from human oocytes, embryos, and fetuses [1, 123]. Furthermore, adult SCs can be extracted and used autologously. Indeed, SCs derived from extra fetal tissues can be isolated after the birth of an individual and kept for potential future use [1]. However, even in well-developed countries, this strategy is not widely used due to its relatively expensive cost, restricted storage capacities, and possible loss of cell viability with storage time, among other factors [1].

**Fig. 5:**
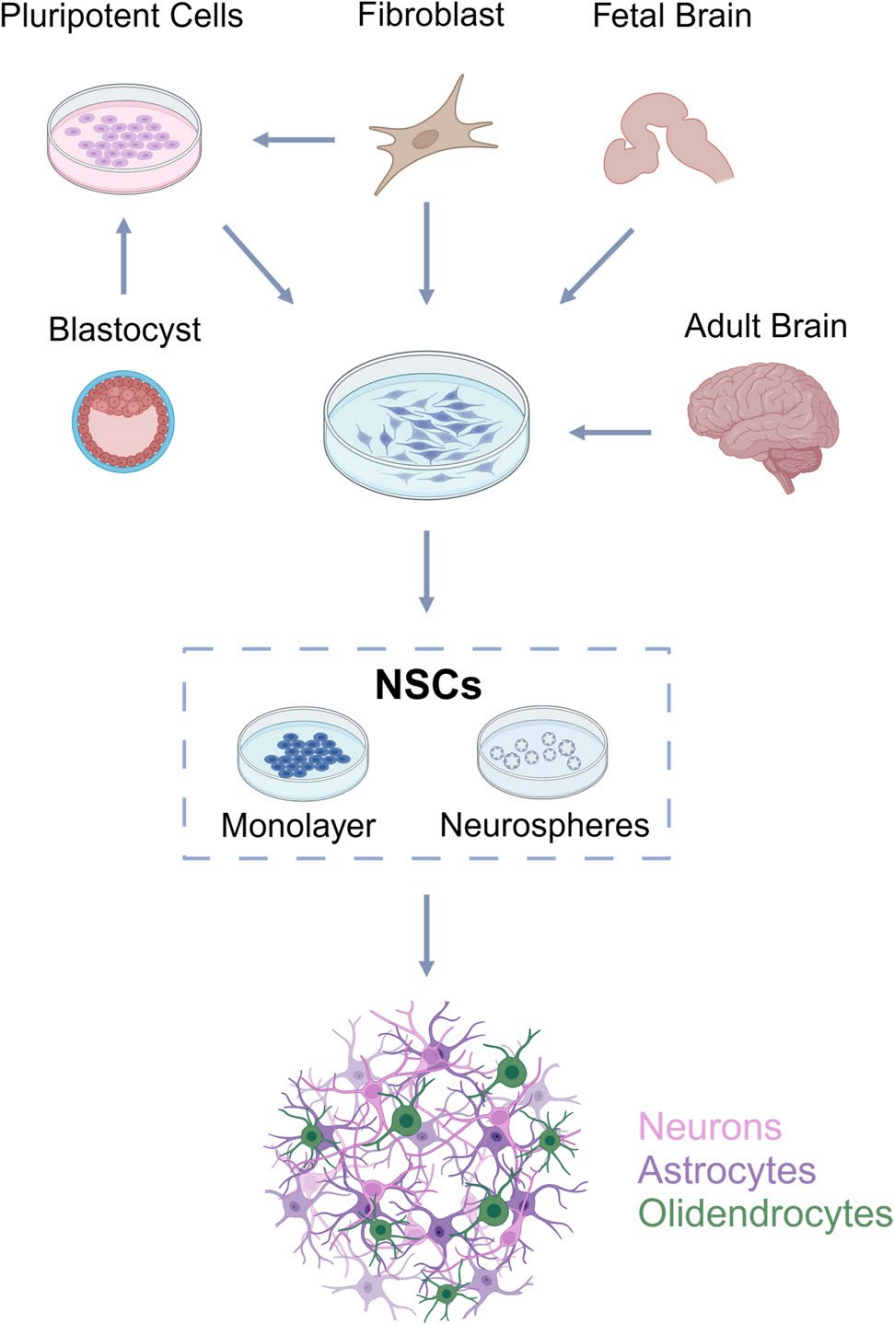
Sources and *in* *vitro* growth protocols for NSC generation and proliferation. There are three main sources of NSCs: direct extraction from primary CNS tissues, differentiation from pluripotent SCs, and trans-differentiation from somatic cells. NSCs can be cultured as neurospheres, which are spherical aggregates of undifferentiated cells that express neural markers, or in monolayer, which is a method of growing cells as a single layer on a flat surface. Created with BioRender.com

Regarding NSCs, they can be derived from three different sources: (1) direct extraction from primary CNS tissues, including fetal brain and adult brain tissue; (2) differentiation from pluripotent SCs, such as embryonic stem cells (ESCs) and iPSCs; and (3) trans-differentiation from somatic cells, for example, skin fibroblasts (Fig. 5) [136]. To obtain and propagate *in* *vitro* NSCs obtained from the first abovementioned method, a serum-free solution containing growth factors such as fibroblast growth factor (FGF) and epidermal growth factor (EGF) is required [136]. Regarding the second method, ESCs are pluripotent cells derived from the blastocyst, a cluster of dividing cells during embryonic development [1]. Besides the ethical issues related to the destruction and manipulation of embryos, these cells are also difficult to control, presenting a high risk of tumorigenesis and the need for immunosuppression [137].

As stated before, iPSCs are obtained by genetic modification of adult postnatal cells. Despite having fewer ethical issues than the latter and fewer immunological problems associated with allogeneic SC transplantation, recent evidence has reported that iPSCs are associated with genomic instability (risk of insertional mutagenesis), immune responses (even to autologous iPSCs), and significant variability among iPSC clones derived from the same donor cells [76, 138]. At last, trans-differentiation of somatic cells into NSCs, also known as lineage reprogramming, involves the transformation of one type of mature somatic cell into another type of mature somatic cell without undergoing an intermediate pluripotent state [136]. Linear reprogramming is considered a promising method for generating NSCs by many in the field, as it eliminates the need for pluripotency and demonstrates remarkable cellular plasticity. However, it is important to acknowledge that this remains an area of active research and debate within the stem cell community.

#### NSC Transplant for Neurological Disease

Regeneration mechanisms are usually triggered by tissue damage. Besides the activation of quiescent NPCs for their participation in the repair process, some other neuroregenerative processes include the regrowth of severed axons, cell renewal, and synaptic plasticity [139]. However, these responsive innate “protective” mechanisms of the CNS are insufficient to fully support the repair of the injured brain cytoarchitecture, being frequently inadequate and transient [140, 141]. To enhance the neurorestorative process while encouraging and supporting the mechanisms mentioned above, the strategy of NPC transplantation has also been developed [142]. This procedure may, indeed, represent one of the most promising approaches in generating new therapy choices for chronic neurological illnesses that are devoid of an effective treatment, including neuropsychiatric disorders. It entails the transplantation of SC-derived cells that have been pre-differentiated *in* *vitro* to different stages of maturation, such as neuroblasts [143].

Although the main goal of NPC transplantation appeared to be the replacement of injured neural cells with newly differentiated cells, this procedure also intends to promote the functional integration of the newly generated cells into the neuronal circuits [130, 140]. Strikingly, the grafted SCs can secrete a wide range of proteins, including growth factors, cytokines, chemokines, metabolites, and bioactive lipids, that also promote tissue repair *in* *vivo* [144]. Some of these molecules have neuroprotective, anti-inflammatory, and immunomodulatory properties crucial for the healing process in the brain. NPCs release all these regenerative molecules to regulate their biology while also directing multiple interactions with the surrounding milieu [144]. This ability of NPCs to adapt their fate and function to the new microenvironment and its needs is called therapeutic plasticity [44]. One of the main determinants of how this therapeutic option is so promising is the fact that NPCs perform a variety of reparative actions within the CNS, including both cell replacement and mediating regeneration by paracrine effects [145–148].

In this therapeutical strategy, one of the first steps is the generation of a subpopulation of NSCs that is well-categorized and can be expanded. For that, the choice of cell source is mainly based on its inherent ability to alter its specified fate to diverse environmental conditions [139]. For example, using cell signals and morphogens involved in CNS development, NSCs can be differentiated directly from pluripotent SCs such as human ESCs and iPSCs [149]. It is important to note that while adult NPCs can give rise to all three neural lineages, their potential for cell replacement is severely limited once they cannot be efficiently directed to most types of neuronal lineages [139]. Several studies using NSC transplantation strategies have already proven that this procedure is a logical and realistic therapeutic approach but only for a limited category of neurological illnesses [150–152], being extremely dependent on the cell type to be replaced as well as the inherent properties of the injured CNS region [139].

The context in which SCs are transplanted is another aspect that critically determines the outcome of the transplant [139]. Therefore, the route for cell administration is critical and highly dependent on the anatomopathological features of the CNS disorder, as the lesion site(s) (focal *versus* multifocal) [139, 153]. While focal CNS disorders, such as PD, favor more direct local cell transplantation (intralesional), systemic transplantation of NPCs (intravenous, intrathecal) might be a more effective strategy in multifocal disorders, such as multiple sclerosis [139]. The stage of the disease is another factor that requires consideration, particularly in diseases with primary inflammation, as NPCs implanted in inflammatory environments have a significantly higher hypothesis of rejection in animals with active neuroinflammation [130]. Interestingly, in animal models of stroke, NSC transplantation has been shown to be beneficial in the sub-acute phase of the disease. This was partially due to the immunomodulatory actions of NSC that triggered a shift from pro-inflammatory M1 microglia toward anti-inflammatory M2, dampening the harmful effects of excessive inflammation [130].

Three main routes may be used to deliver NSCs *in* *vivo*: intravenous, intraparenchymal, or intra-cerebroventricular via lumbar puncture injection. Research performed in animals has shown that after intraparenchymal delivery, NSCs migrate and spread along the corpus callosum or can be driven by tissue-specific disease factors emanating from injured tissue [154]. When injected intravenously, NPCs cross the inflamed blood–brain barrier (BBB) and reach the demyelinating areas of the CNS in animal models of multiple sclerosis, where they exert therapeutic actions [154].

Curiously, there has also been some interest in the intranasal application (INA) of SCs due to their less invasive nature [155]. Indeed, in some situations, INA has some advantages over other routes of administration, which include the delivery of a higher amount of the therapeutic compound in the CNS, the bypass of the first-pass metabolism, and the fact of being non-invasive and easy to administer, enabling repeated administration if required. Moreover, INA reduces adverse effects since the therapeutic compound does not reach other healthy organs [155]. Nevertheless, there are still some hurdles to be addressed before translation from animal experiments to clinical application of INA in humans. One such challenge is overcoming the BBB, which significantly limits the efficacy of cell delivery to the CNS, particularly through non-invasive methods. Additionally, this method exhibits a lack of reliability due to the inconsistent distribution of cells following intranasal injection, a variability that is observed even within the same animal model [155].

The first CNS disorders seen as potential targets for this SC therapy were neurodegenerative disorders, such as PD and Alzheimer’s disease, and cerebrovascular diseases, such as ischemic stroke and spinal cord injury, i.e., disorders essentially characterized by neurodegeneration and cell loss [76]. Concerning neurodegenerative diseases, the available therapies attenuate symptoms but fail to cure the illness, being ineffective in rescuing or regenerating cellular function [156]. This limitation in current therapies has driven the development of several preclinical and clinical trials focused on alternative approaches. Accordingly, several preclinical and clinical trials were recently developed or are ongoing, reporting the use of NSC transplantation, in successful cell-based therapies for different diseases (Table 1).

**Table 1:** List of clinical trials using NPC/NSC in non-pediatric subjects

Disease	Title and NCT number	Cell type	Trial phase	No. of patients	Status
Amyotrophic lateral sclerosis	Human neural stem cell transplantation in amyotrophic lateral sclerosis (ALS) (hNSCALS) NCT01640067	Human fetal NSCs	Phase 1	18	Completed
Amyotrophic lateral sclerosis	Dose escalation and safety study of human spinal cord-derived neural stem cell transplantation for the treatment of amyotrophic lateral sclerosis NCT01730716	Human NSCs	Phase 2	18	Unknown status
Brain tumors	Genetically modified neural stem cells, flucytosine, and leucovorin for treating patients with recurrent high-grade gliomas NCT02015819	Human NSCs	Phase 1	16	Completed
Brain tumors	Neural stem cell-based virotherapy of newly diagnosed malignant glioma NCT03072134	Induced NSCs	Phase 1	12	Completed
Brain tumors	Repeated neural stem cell-based virotherapy for newly diagnosed high-grade glioma NCT06169280	NSCs conditionally replicative adenovirus (CRAd)-survivin (S)-protomer (p)k7 (NSC-CRAd-S-pk7)	Phase 1	20	Recruiting
Diabetes mellitus, type 2; diabetes mellitus type 2 in obese and nonobese; obesity; cognitive dysfunction; metabolism disorder	From skin fibroblasts to NSCs to investigate *in* *vitro* the impact of diabetes on adult neurogenesis NCT05755321	Induced NPCs	Phase 1	40	Active, not recruiting
Ischemic stroke	A safety and tolerability study of NSCs (NR1) in subjects with chronic ischemic subcortical stroke (ISS) NCT04631406	Human NSCs	Phase 1 Phase 2	30	Recruiting
Ischemic stroke	Intracerebral transplantation of NSCs for the treatment of ischemic stroke NCT03296618	Human NSCs	Phase 1	18	Active, not recruiting
Parkinson’s disease	A study to evaluate the safety and efficacy of human NSCs for Parkinson’s disease patient (hNSCPD) NCT03128450	Human NSCs	Phase 2 Phase 3	12	Unknown status
Parkinson’s disease	A study on the treatment of Parkinson’s disease with autologous NSCs NCT03815071	Induced NSCs	Early Phase 1	10	Active, not recruiting
Parkinson’s disease	Safety and efficacy study of human ESC-derived neural precursor cells in the treatment of Parkinson’s disease NCT03119636	Human ESC-derived neural precursor cells	Phase 1 Phase 2	50	Unknown status
Parkinson’s disease	Study to evaluate the safety and efficacy of ESC-derived dopamine progenitor cell therapy in PD patients NCT05887466	High-purity midbrain dopaminergic progenitor cells derived from human embryonic stem cells (hESCs)	Phase 1/2a	12	Active, not recruiting
Parkinson’s disease	Phase 1 safety and tolerability study of MSK-DA01 cell therapy for advanced Parkinson’s disease NCT04802733	Human embryonic stem cell-derived midbrain dopamine neuron cells (MSK-DA01)	Phase 1	12	Completed
Parkinson’s disease	Continued evaluation of patients with Parkinson’s disease who previously received BRT-DA01 NCT05897957	Human embryonic stem cell-derived midbrain dopaminergic neurons (BRT-DA01)	Phase 1	12	Enrolling by invitation
Progressive multiple sclerosis	NSC transplantation in multiple sclerosis patients (STEMS) NCT03269071	Human foetal-derived NSCs	Phase 1	4	Completed
Secondary-progressive multiple sclerosis	Safety study of human NSC injections for secondary-progressive multiple sclerosis patients (NSC-SPMS) NCT03269071	Human NSCs	Phase 1	24	Completed
Spinal cord injury	Safety study of human spinal cord-derived NSC transplantation for the treatment of chronic SCI (SCI) NCT01772810	Human NSCs, spinal cord-derived	Phase 1	8	Unknown status
Spinal cord injury	NeuroRegen Scaffold™ combined with stem cells for chronic spinal cord injury repair NCT02688049	Human NSCs	Phase 1 Phase 2	30	Unknown status

*NSCs*, neural stem cells; *NPCs*, neural progenitor cells; *ESC*, embryonic stem cell

Curiously, PD is one of the best-suited neurodegenerative diseases for the use of SC-based therapy [157], being characterized by extensive depletion of dopaminergic neurons in a specific area, the substantia nigra. Therefore, clinicians hope to replace the lost dopaminergic neurons with SC differentiated into the dopaminergic lineage. Several clinical trials have already proven that transplant of fetal midbrain tissues can help stratum reinnervation, relieve neurological symptoms, and restore motor functions in patients with PD [133, 158–160]. However, the survival of the transplanted fetal mesencephalic cells is not sufficient [161]. As an alternative, pluripotent SCs, such as ESCs and iPSCs, can be differentiated into neurons with dopaminergic properties. Indeed, dopaminergic neurons have been efficiently engrafted *in* *vivo* after transplantation and have functionally recovered motor deficits in animal models of PD [162].

Alzheimer’s disease (AD) is the most prevalent neurodegenerative disease associated with dementia. Studies have shown that transplanted NSCs in animal models of AD are capable of differentiating into mature cell types, improving their cognitive behavior [163]. In fact, a study performed in a transgenic mouse model of Alzheimer’s disease showed that individual memory function can be recovered by the release of the brain-derived neurotrophic factor and the cell repair associated with the transplant [164, 165]. However, it is still not clear the exact mechanism by which transplanted cells exert their beneficial effects through neuronal replacement or their paracrine effects [166]. Another interesting approach for AD was the transplantation of NSCs as a vehicle to deliver potential therapeutic agents, such as neprilysin, an Aβ-degrading protease that reduces amyloid plaques in AD mice [165–167].

Interestingly, NSC transplantation has also been proven a valued therapeutic strategy for ischemic stroke. Here, the abrupt and near-total interruption of cerebral blood flow first results in reversible tissue function loss that ultimately culminates in the loss of neurons and supportive structures, as ischemia is established [168]. One promising strategy for stimulating neurogenesis and facilitating neural recovery after ischemic stroke is indeed SC therapy [118]. For example, the transplantation of human ESC-derived NPCs implanted in animal models of stroke was capable of inducing neural differentiation and improving functional recovery of the animals [169]. Moreover, preclinical trials demonstrated that transplanted NSCs reduced cell death and inflammation near the graft while also stimulating blood vessel growth (angiogenesis) [169]. This approach also increased the proliferation and neuronal differentiation of endogenous NSCs in rodents, primates, and humans [170]. Here, the implanted NSCs survived intracerebral transplantation and differentiated into mature neurons, being able to integrate the host neurocircuitry. Strongly, this integration allowed them to promote morphological and electrophysiological recovery after stroke, even several months later [170].

Thus, following the success of NSC transplantation, the activation of endogenous NPCs for neural tissue regeneration following ischemic injury has also emerged as a potential therapeutic approach [170]. Curiously, the administration of growth factors, such as EGF and FGF, has also been shown to stimulate the mobilization of endogenous NPCs and restore the hippocampal circuitry and synaptic function after ischemia [171].

Nevertheless, it is important to note that despite the potential of SC-based strategy for all mentioned neurological conditions, several challenging and clinical concerns must be solved for its clinical translation [153]. Teratoma formation or abnormal cell growth, immunological rejection and inflammation, and undesired cell differentiation phenotypes are all potential severe safety hazards of this type of therapy [149]. Thus, every stage of the process requires meticulous attention, from cell origin to *in* *vitro* cell growth, differentiation, and modification to final administration to patients [149]. It is, therefore, crucial to establish standardized differentiation/selection protocols to produce cells with reproducible, predictable, and optimal gene and protein expression, as well as stringent quality control to successfully implement this therapy in clinics [149].

#### NSCs for Neurotoxicology Tests

NSCs have proven invaluable for neurotoxicity testing due to their early differentiation in development and the ease of replicating this process *in* *vitro* [170]. During prenatal life, a small number of cells need to give rise to the human brain and all its complexity. During this period of development and taking into account all the considerable changes in cell quantity, overall size, and shape, the CNS is intrinsically more vulnerable to chemical exposure [170, 172]. From this idea, the requirement for ad hoc pharmacological testing is well established [170], and the demand for suitable tools to study the adverse effects of chemical substances and physical agents on the different stages of brain development has led researchers to look for other alternatives in comparison with the usual animal studies [170, 173].

NSCs, particularly when derived from hiPSCs, offer a viable and ethical option for studying neurotoxicity. Human *in* *vitro* neuronal cultures produced from NSCs or brain fetal NPCs cultured as neurospheres can accurately replicate critical brain development events such as proliferation, apoptosis, migration, and differentiation [174]. To avoid the ethical issues relating to the use of human embryonic or fetal-derived tissues, the application of human iPSC-derived cultures of mixed neuronal and glial cells also emerged. These cells can serve as systems for toxicity testing, particularly for the assessment of different pathways involved in neurotoxicity [175].

A huge effort has been put into recreating the brain development process more accurately better to analyze the real impact of exposure to toxic chemicals. Thus, to introduce the immune component into the neuroglia culture, microglia-like cells and brain endothelial cells have been grown from hiPSCs [176] to mimic the activity of the BBB *in* *vitro* [177]. However, there is still a long way to go before replacing *in* *vivo* animal laboratory tests with *in* *vitro* iPSC-derived cell population assays in toxicological testing [172]. First, there is a need to establish procedures for generating differentiated cells that are reproducible and trustworthy while ensuring the right quantity and purity of produced cells [172]. At last, reliable and valid assays must be generated for a wide range of chemicals [172].

#### iPSC-derived NSCs for Disease Modeling and Drug Discovery

iPSCs have revolutionized the field of neurological disorders by enabling the derivation of patient-specific NSCs for disease modeling and drug discovery. These NSCs can develop into various neural cell types, providing a more accurate representation of human diseases. iPSCs are generated from differentiated mature cells whose expression of specific genes is manipulated. This process results in pluripotency restoration [178, 179], yielding iPSCs unique characteristics such as processing a patient’s genetic background and being able to differentiate into various cell types. This process depends on the manipulation of several signaling pathways. In the case of the iPSC-derived NSCs, inhibition of the Sma-and Mad-related protein (SMAD) signaling is crucial. This is accomplished by exposing iPSCs to SB431542 and Noggin, both of which are inhibitors of SMAD upstream pathways *TGF-β* and *BMP-4*, respectively*.* The use of iPSCs to generate NSCs offers several advantages in relation to the use of embryonic stem cells (ESCs), such as the maintenance of a specific genetic background. Consequentially, iPSC-derived NSCs can be used for more realistic disease modeling and drug testing [179, 180].

Drug discovery is a complex process that requires knowledge of the pathological mechanisms responsible for a certain disease. Disease models are key in the stages of lead drug discovery and preclinical development. Thus far, animal models have proven to be quite helpful in the identification of human disease etiologies and new targets for drug screening, as well as in the evaluation of drug efficacy before clinical trials [181]. Nevertheless, these models do not accurately represent human diseases for obvious reasons. As an example, even though numerous transgenic mouse models of AD have been developed, none of them have been able to fully reproduce the pathology of the human illness. Hence, there was a clear need to invest in human disease modeling platforms to support and complement the studies that were performed on animal models [182].

In the field of neurological disorders, recent advances with iPSCs have enabled disease modeling with patient-derived neural cells. These differentiated cells can be used to develop disease-relevant assays for drug screening, offering a new strategy to model and study diseases that occur casually, i.e., that appear without a known cause, as well as genetic diseases. This concept is quite valuable within neuronal disorders, as many occur sporadically, such as AD, with 95% of patients showing sporadic onset. Thus, a new path for disease modeling and drug discovery has been opened by the iPSC technology since patient-derived iPSCs and their respective NSCs represent a more relevant disease system in the proper setting.

Of note, since iPSCs can be easily obtained from patients with neurological disorders and differentiated into disease-relevant cells, such as NSCs and neurons. The use of iPSC-based disease modeling is already prevalent for the investigation of disorders caused by a single gene mutation or having an early onset. Moreover, due to the relatively low level of maturation of iPSC-derived NSCs, there is a higher degree of confidence that the characteristics of differentiated cells represent a suitable model for diseases with an early onset. On the other hand, in diseases with late onset, cellular aging is the major player in disease pathogenesis. To overcome this drawback, induced cellular aging has been employed. One possible method to induce senescence is to expose the differentiated cells to cellular stressors, such as compounds that affect mitochondrial function or protein degradation [182]. Another approach is to overexpress gene products that cause premature aging. However, it is yet unknown whether these techniques can cause cellular aging through a mechanism that resembles normal aging [182]. In contrast, the direct reprogramming approach that entails the direct conversion of human fibroblasts into other lineage-specific cells, such as neurons, does not eliminate cellular aging markers [183]. In fact, neurons derived from aged fibroblasts through direct reprogramming have been demonstrated to preserve cellular age [184], providing a better cellular model to examine age-related disorders.

The first step to produce a disease model using hiPSCs is to generate these cells with the disease-causing mutation(s). Then, these cells are differentiated into disease-relevant cell types, such as NSCs, and the resulting cells are used to determine the pathogenic pathways and the cause of the disease. As aforementioned, NSCs and NPCs can be quickly generated from iPSCs in large quantities and with high levels of reproducibility. NSCs and NPCs may then exhibit the relevant disease phenotypes, being employed as disease models for compound screening and efficacy studies. Indeed, since neurons can then be generated from NSCs or iPSCs differentiation, iPSCs can be differentiated into specific neuron types, such as dopaminergic neurons or astrocytes and oligodendrocytes [182]. This renders iPSC-derived NSCs crucial for the potential development of several disease models. For example, by exposing NSCs derived from patients with PD to sonic hedgehog protein and fibroblast growth factor-8, it is possible to accurately generate a patient-specific model for PD [185]. Through this methodology, it is possible to study, *in* *vitro*, cells that reflect the real pathophysiology of a disease rather than approximations such as animal models.

As patient-derived iPSC models share the same genetic background and illness phenotype of the patients, they are more suited for phenotypic-based drug discovery [181]. Furthermore, several cell-level phenotypes were identified in patient-specific iPSCs across several diseases, enabling the reproduction of disease phenotypes and pathologies in a culture dish. These include impaired motor neurons from spinal muscular atrophy patient-derived iPSCs and decreased neuronal connectivity, neurite formation, and synaptic protein expression in neurons from schizophrenia patient-derived iPSCs [186].

Therefore, phenotypic screening has recently gained popularity in the drug discovery process as target-based drug discovery slowly loses its strength [187]. The advances made in iPSC technology and their derived NSCs have enormously contributed to the reappearance of phenotypic screening. Indeed, their scalability of production facilitates the development of assays, while their pluripotency potential ultimately allows differentiation into a variety of disease-relevant cell types, particularly the ones with difficult access, such as neurons. Importantly, the relevance of the chosen phenotype as a readout for drug screen can be tested by a gene editing approach if the gene responsible for the disease phenotypes is known and can be further corroborated in patient samples and/or animal models [182].

Thus, numerous drug screens for prospective therapeutic agents have been performed and discovered, respectively, either using phenotypic or target-oriented screening methods through the employment of models derived from hiPSCs. One example is the research conducted by Bright and colleagues [188]. The authors made effective use of a patient-derived iPSC model of AD by detecting a disease-relevant protein, extracellular tau (eTau), in the conditioned medium of iPSC-derived cortical neurons of AD patients, generating a therapeutic antibody against eTau [188]. This disease-relevant protein, which induces neuronal hyperactivity and increases amyloid-β production, would not have been detected without using the human iPSC model [182].

It is, therefore, undeniable that the application of iPSCs in modeling neurological diseases is an important alternative to animal disease models for drug discovery and development, with more than 1000 compounds evaluated and several clinical candidates already identified. However, there are still some limitations in the use of these cells. This approach requires significant time to generate disease-relevant cell types from iPSCs, such as NSCs and their mature counterparts, which may compromise the quality and stability of these cells. Hence, there is a demand for faster and more reliable differentiation methods that yield higher maturity and purity [182, 186]. Other potential limitations are the absence of environmental factors *in* *vitro* with a significant role in certain neurologic disorders [181, 189], and the possible insufficient representation of iPSC-derived neurons for different developmental stages of the fetal brain [181]. Nevertheless, and despite these challenges, the field of iPSC research is rapidly evolving, with scientists around the world working tirelessly to overcome all weaknesses.

Recent advancements in technology and methodology are paving the way for more efficient differentiation processes. As an example, the development of 3D culture systems is expected to provide a more accurate representation of the *in* *vivo* environment. Furthermore, the incorporation of patient-specific environmental factors and lifestyle habits, such as diet- and physical activity-related systemic factors, into *in* *vitro* models could offer unprecedented insights into the pathogenesis of neurologic disorders.

It is also important to note that although these systems allowed a better understanding of the mechanisms underlying modeled diseases, they do not mimic the complex multicellular environments in which human disease occurs. In the human body, cells interact with extracellular matrices, tissues, organ systems, and pathogens. Therefore, more advanced iPSC-based differentiation systems are being developed to accurately reproduce human tissue-level and organ-level dysfunction [186].

**Fig. 6:**
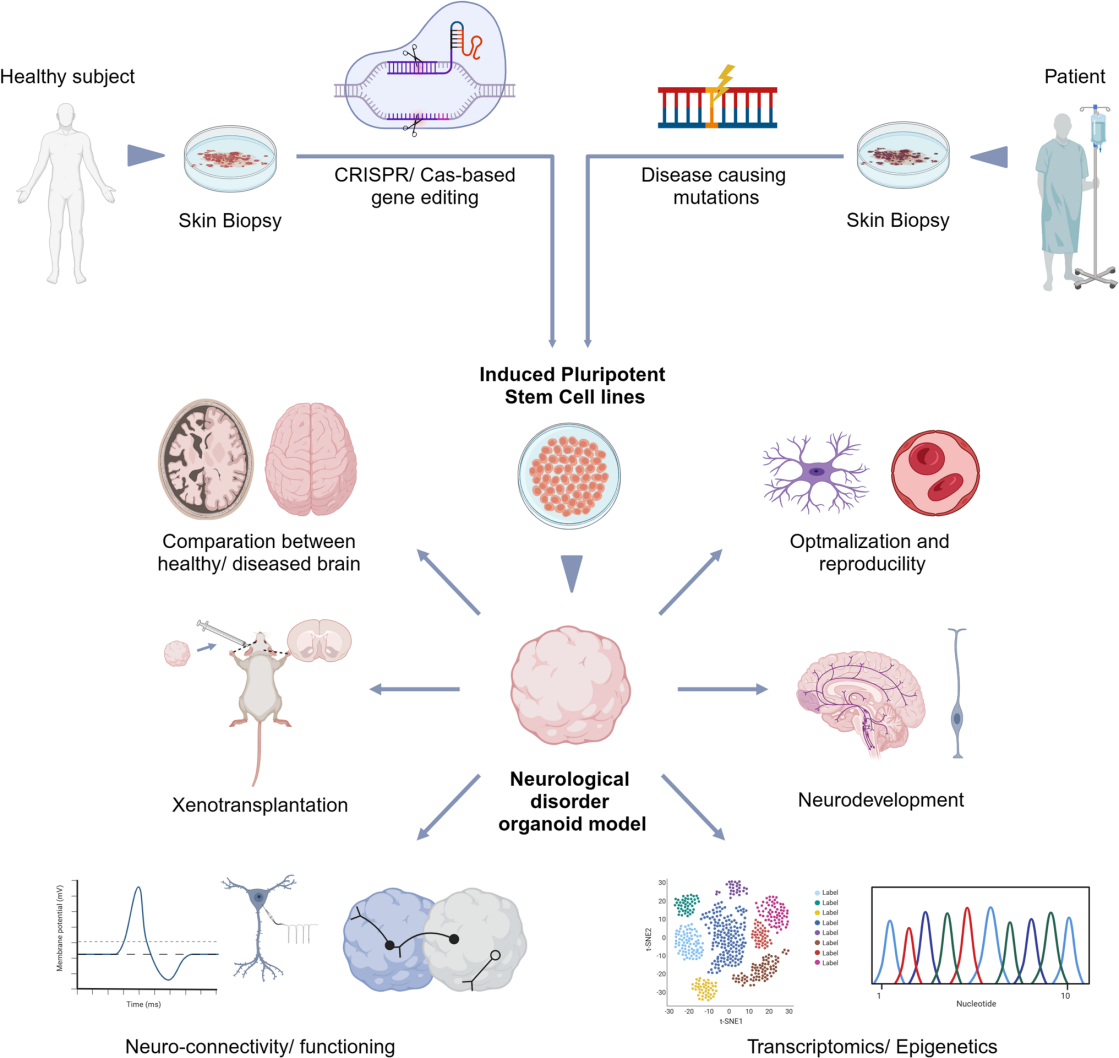
Diagrammatic representation of the generation and applications of organoid models for neurological disorders. Brain organoids can be generated from iPSCs. These platforms can mimic the pathological features of various neurological disorders if derived from either genetically modified cells with disease-related mutations or cells harvested from patients with these disorders. This makes them a helpful tool in studying their mechanisms and features. They can also be used in the study of neurodevelopment, being able to recapitulate the dynamic spatiotemporal process of early brain development. Furthermore, brain organoids can also be used as a platform to screen and test potential drugs for their efficacy and safety. Adapted from [215]. Created with BioRender.com

iPSC-derived organoids are an example of those advanced systems. Organoids, also referred to as “organs in a dish,” are 3D multicellular structures derived from SCs that undergo differentiation and self-organization to mimic the morphological characteristics and cellular interactions of mature tissues [190]. They enable analysis of disease pathology in a developmentally relevant spatial–temporal context and have the potential to provide a drug response at the level of an organ rather than at the level of single cells. Recently, they have already been applied in modeling human organ development and diseases, screening therapeutic compounds, and cell transplantation [182]. The 3D human neural organoids are, therefore, valuable platforms to study the distinctive anatomical and physiological features of the human nervous system as well as human neuronal fate specification and disease pathogenesis [191], having already been employed in the modeling of neurodevelopmental, neuropsychiatric, and neurodegenerative disorders, such as microcephaly, Miller–Dieker syndrome, and AD [192]. As iPSC-derived organoids enable *in* *vitro* and *in* *vivo* studies, they have the strong potential to bridge preclinical and clinical trials, being able to simulate aspects of human physiology more accurately than animal models (Fig. 6) [192, 193]. However, once again, some challenges are still limiting the full application of organoid technology in drug discovery. For example, the intra-organoid and inter-organoid cellular heterogeneity, the restricted scalability, the low reproducibility across protocols, and the variable degree of maturity still represent major obstacles [192]. So far, organoid-based research has been predominantly conducted in academic settings, with only a few companies actively investing in the technology for scalable production [192]. Nevertheless, the demand for commercial organoids will probably increase in the next few years [192].

## Conclusions and Future Perspectives

SC research is certainly one of the most auspicious and appealing fields in neurobiology, as it offers the possibility of generating new neurons and glia for the treatment and comprehension of several neurological disorders. Here, we reviewed the physiological role of NSCs in sustaining brain homeostasis and restoring brain damage, as well as some of the current advances and challenges of their use in neurological disease modeling, drug screening, and neurotoxicity testing. These possibilities were only achieved with the significant progress made in SC research, especially with the development of new techniques and technologies for isolating, culturing, manipulating, and transplanting NSCs and their derivatives.

Despite those advances, there are still several limitations and open questions that remain unanswered. Ethical issues, immunological compatibility, tumorigenicity, poor differentiation efficiency [149], low survival rate of the cells [194], and lack of specific markers are obstacles that need to be tackled [195]. A further limitation regarding SC-based therapies is also the limited accessibility of these cells for some patients [196]. Indeed, there are some conflicting and exclusion criteria for patients with certain conditions, such as poor health or major organ dysfunction, i.e., patients who cannot withstand surgery or cell therapy procedures [196].

Thus, future research in this field must focus on addressing these challenges and improving the quality and safety of NSC-based applications. Some of the possible paths might include the optimization of the culture conditions and genetic manipulation of NSCs to get viable cell cultures [197], the documentation of new sources and subtypes of NSCs to offer more options and diversity for the generation and differentiation of neural cells, and the development of new methods for tracking and imaging NSCs *in* *vivo*, providing better monitoring of transplanted NSCs and better evaluation of their efficacy and safety [198]. The establishment of standardized protocols and guidelines for NSC generation and transplantation, as well as rigorous quality control, are also crucial [196].

Regarding NSC transplantation, the clinical outcome and long-term safety of this therapy in patients remain uncertain [149]. A key challenge is to ensure that the transplanted cells can indeed integrate into the host brain, form functional neural networks, and restore the neurological functions impaired by the disease [196]. Additional preclinical studies are required to elucidate the molecular and cellular mechanisms underlying the fate and function of NSCs, as well as their therapeutic effects in disease models [149]. This will certainly provide a stronger and more accurate basis for their clinical translation.

Despite these challenges, the study of the therapeutical properties of the NSC secretome and the factors that influence its function and composition is a promising approach. NSCs are in close contact with the vasculature in both niches and with the CSF in the SVZ. Furthermore, they are known to influence their surroundings through their secretome. This renders NSCs as perfect players in detecting and integrating systemic cues into the CNS while influencing neurogenesis. Indeed, processes such as proliferation and differentiation depend on the overall homeostasis of the niche, which is often affected in disease states [199]. Moreover, obesity and depression are associated with low-grade systemic inflammation [200, 201] that disrupts the BBB, inducing an inflammatory state in the brain and pushing the resident microglia towards activation [202], leading to loss of homeostasis in the NSC niche. In turn, activated microglia release pro-inflammatory cytokines that disrupt NSC proliferation [203], differentiation [204], and neurogenesis overall [205]. Interestingly, the NSC secretome is rich in anti-inflammatory factors such as IL-10 [206], prostaglandin 2, and oxid nitric [207]. Moreover, extracellular vesicles, a key component of the NSC secretome, often have anti-inflammation CD81 receptors on their surface [208]. These extracellular vesicles are also known for mitochondrial transfer between NSCs and other cell types, a process that shapes macrophage metabolism, leading to decreased inflammatory activity [209].

Curiously, the development of several neurodegenerative diseases has been related to the age-associated decline of neurogenesis. This process results from a diminished capacity to undergo neurogenesis and to secrete a supporting secretome [199]. Indeed, due to their exposure to systemic cues, NSCs are also subjected to whole-body changes that occur throughout aging. One of the most prominent ones is the development of a systemic pro-inflammatory state designated as inflammaging, which is associated with high levels of pro-inflammatory molecules [210]. This results in the development of a pro-inflammatory secretome that partially contributes to age-associated cognitive decline [106]. An understanding of the factors that ameliorate this process may prove crucial to prevent this type of disease. These findings translate into an opportunity to modulate NSC secretome properties through exposure to therapeutic targets and, therefore, to treat or even prevent inflammatory low-grade associated diseases. A prominent example of this approach is the use of antidepressants. Although they were not developed specifically for neurogenesis enhancement, the actions of the antidepressant fluoxetine result, at least partially, from its neurogenic effect [211]. Therefore, we can think of fluoxetine as a neurogenesis-modulating drug used to treat depression. The use of proteins to modulate NSC action is an elegant approach to overcome the difficult challenges associated with the development of SC direct use-based therapeutics. Another interesting advantage of this approach is the possibility of non-invasive delivery of such targets via oral tablets or even intranasal delivery.

In addition to disease modeling and prevention via the NSC secretome, another promising strategy may be the characterization of the NSC secretome and its therapeutic capabilities beyond neurogenesis. Indeed, a significative part of the beneficial effects of the NSC transplants are a consequence of supporting factors secreted by exogenous NSCs rather than the NSCs *per si* [212]. Even if the transplanted NSCs survive and integrate, the NSC secretome is undoubtedly responsible for some of the therapeutical effects of NSC transplants. This opens the door to several factors, such as IGF-II and BFNF, to serve as therapeutic options. This strategy overcomes some of the problems associated with NSC transplants, namely the tumorigenicity of SCs, undesired differentiation, and limited graft survival, among others. Additionally, it enables easier production methods with higher reproducibility, safety, and efficacy.

At last, it is important to recognize that studying human neuroplasticity at the molecular level is still remarkably challenging due to the huge complexity of the human brain and the difficulty of collecting live human neural tissue and/or cells. Fortunately, breakthroughs using molecular tools and stem cell models are on the horizon. They are emerging to resemble the individual physiology of the human brain and help to understand the mechanisms of neurological diseases. These innovative approaches will certainly revolutionize the next decades of the NSC field.

## Data Availability

No datasets were generated or analysed during the current study.
