# Correction: Sustaining Brain Youth by Neural Stem Cells: Physiological and Therapeutic Perspectives

**DOI:** 10.1007/s12035-025-05260-2

**Published:** 2025-07-30

**Authors:** Matilde Santos, João A. Ferreira Moreira, Sónia Sá Santos, Susana Solá

**Affiliations:** Research Institute for Medicines (iMed.ULisboa), Faculty of Pharmacy, Universidade de Lisboa, 1649-003 Lisbon, Portugal

**Correction: Molecular Neurobiology (2025) 62:8222-8247**

10.1007/s12035-025-04774-z

In the original article [1], there was a mistake in the Figure legends 1, 2, 3, 4 and 6 as published. The attribution “Adapted from [respective reference]” and the references [213], [214] and [215] were omitted due to an oversight. The corrected figure legends, now including the appropriate references, are provided below.

The authors apologize for this error and state that it does not affect the scientific conclusions of the article in any way.

The original article has been updated.

Updated Figure Legends

Fig. 1 - The "novel" and "classical" adult neurogenic zones, as well as the major cellular stages of neurogenesis, depicted schematically: Schematic representation of the different brain regions, classic – the hippocampus and subventricular zone - and “novel” - the prefrontal cortex, hypothalamus, striatum, amygdala, and piriform cortex - where progenitor cells are generated. Experiments have shown that progenitor cells can stray from the conventional path in the DG and SVZ and differentiate and mature in diverse brain regions, including the prefrontal cortex, striatum, substantia nigra, and amygdala, which are referred to as "novel neurogenic zones." CA3, cornu ammonis 3 (region of the hippocampus). Adapted from [213]. Created with BioRender.com.

Fig. 2 – Regulation of neurogenesis by local circuit factors: All stages of neurogenesis (proliferation, differentiation, and survival) are a target of regulation by network factors. Local GABA appears to suppress proliferation while inducing differentiation and survival. Glutamate is necessary for proper survival as well. Modulatory neurotransmitters appear to regulate different parts of the process; for instance, serotonin induces proliferation, whereas acetylcholine is necessary for proper maturation and survival. The effects of the other major neuromodulatory systems [norepinephrine, dopamine] on neurogenesis are less well understood. CA3, cornu ammonis 3 (region of the hippocampus); GABA, gamma-aminobutyric acid; 5HT, serotonin; ACh, acetylcholine; NE, norepinephrine; DA, dopamine. Adapted from [4]. Created with BioRender.com.

Fig. 3 – Overall goals of regenerative medicine: Regenerative medicine is a field of research that aims to restore or replace damaged human cells, tissues, or organs and to positively control the adult stem cell compartment so that stem cells remain robust throughout adulthood to improve health and quality of life. SCs, stem cells. Adapted from [123]. Created with BioRender.com.

Fig. 4 – Molecular and cellular changes associated with neural aging: Several basic homeostatic functions in the brain become altered through aging. These include genomic instability, mitochondrial dysfunction, deregulated nutrient sensing, epigenetic alteration, altered cell proliferative capacity and intercellular communication, loss of proteostasis, among others. Adapted from [214]. Created with BioRender.com.

Fig. 6 - Diagrammatic representation of the generation and applications of organoid models for neurological disorders: Brain organoids can be generated from iPSCs. These platforms can mimic the pathological features of various neurological disorders, if derived from either genetically modified cells with disease-related mutations or cells harvested from patients with these disorders. This makes them a helpful tool in studying their mechanisms and features. They can also be used in the study of neurodevelopment, being able to recapitulate the dynamic spatiotemporal process of early brain development. Furthermore, brain organoids can also be used as a platform to screen and test potential drugs for their efficacy and safety. Adapted from [215]. Created with BioRender.com.

References

[1] Santos, M., Moreira, J. A., Sá Santos, S., Solá, S. (2025) Sustaining brain youth by neural stem cells: physiological and therapeutic perspectives. Mol Neurobiol, 62 (7), 8222-8247. doi: 10.1007/s12035-025-04774-z.

[213] Vaz, A., Ribeiro, I., & Pinto, L. (2022). Frontiers in Neurogenesis. Cells, 11(22), 3567. https://doi.org/10.3390/cells11223567

[214] Jin, M., Cai, S. Q. (2023). Mechanisms underlying brain aging under normal and pathological conditions. Neurosci Bull, 39 (2), 303-314. doi: 10.1007/s12264-022-00969-9.

[215] Gazerani, P. (2023). Human brain organoids in migraine research: pathogenesis and drug development. Int. J. Mol. Sci., 24 (4), 3113. doi: 10.3390/ijms24043113.

